# Octa­methyl­bis­(μ_2_-2-methyl­benzoato-κ^2^ *O*:*O*′)bis­(2-methyl­benzoato-κ*O*)di-μ_3_-oxido-tetra­tin(IV)

**DOI:** 10.1107/S1600536810036512

**Published:** 2010-09-18

**Authors:** Muhammad Danish, Sabiha Ghafoor, M. Nawaz Tahir, Nazir Ahmad, Masood Hamid

**Affiliations:** a Department of Chemistry, University of Sargodha, Sargodha, Pakistan; b Department of Physics, University of Sargodha, Sargodha, Pakistan

## Abstract

The title compound, [Sn_4_(CH_3_)_8_(C_8_H_7_O_2_)_4_O_2_], is a distann­oxane derivative of 2-methyl­benzoic acid. The crystal structure is composed of centrosymmetric dimers lying about inversion centres. Both independent Sn atoms adopt distorted trigonal-bipyramidal SnC_2_O_3_ coordination geometries with the basal planes consisting of two C-atoms from the methyl groups and a bridging O atom. The Sn—C and Sn—O bond lengths lie in the ranges 2.090 (2)–2.104 (3) and 2.0241 (14)–2.2561 (15) Å, respectively. The central four-membered planar Sn_2_O_2_ ring [Sn⋯Sn distance = 3.2993 (2) Å] makes dihedral angles of 5.43 (11) and 59.50 (7)° with the methyl­phenyl groups, which are themselves oriented at a dihedral angle of 61.38 (8)°. Besides weak C—H⋯O and C—H⋯π inter­actions, the packing mainly features van der Waals forces between the mol­ecules.

## Related literature

For distannoxanes, see: Amini *et al.* (2002 ▶); Danish *et al.* (2009 ▶).
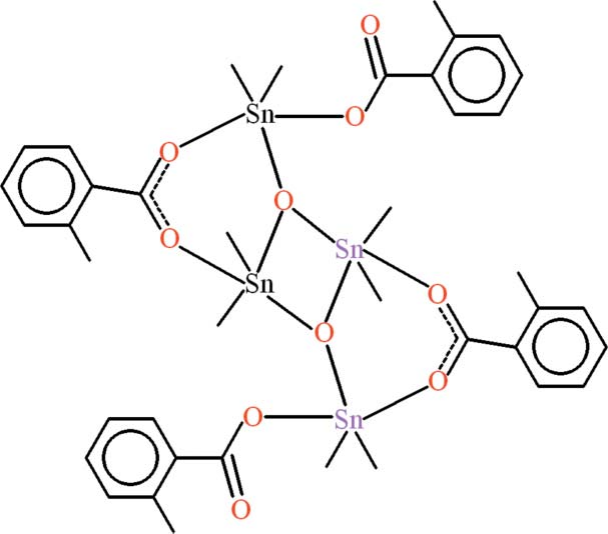


## Experimental

### 

#### Crystal data

[Sn_4_(CH_3_)_8_(C_8_H_7_O_2_)_4_O_2_]
*M* *_r_* = 1167.66
Triclinic, 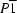

*a* = 10.0413 (2) Å
*b* = 10.1280 (2) Å
*c* = 12.0910 (3) Å
α = 83.300 (1)°
β = 72.850 (2)°
γ = 71.876 (1)°
*V* = 1116.24 (4) Å^3^
*Z* = 1
Mo *K*α radiation
μ = 2.26 mm^−1^
*T* = 296 K
0.30 × 0.26 × 0.23 mm

#### Data collection

Bruker APEXII CCD diffractometer
Absorption correction: multi-scan (*SADABS*; Bruker, 2005 ▶) *T*_min_ = 0.514, *T*_max_ = 0.593
18376 measured reflections
5470 independent reflections
4786 reflections with *I* > 2σ(*I*)
*R*_int_ = 0.024

#### Refinement

*R*[*F* ^2^ > 2σ(*F* ^2^)] = 0.018
*wR*(*F* ^2^) = 0.049
*S* = 1.09
5470 reflections
250 parameters
H-atom parameters constrained
Δρ_max_ = 0.43 e Å^−3^
Δρ_min_ = −0.40 e Å^−3^

### 

Data collection: *APEX2* (Bruker, 2009 ▶); cell refinement: *SAINT* (Bruker, 2009 ▶); data reduction: *SAINT*; program(s) used to solve structure: *SHELXS97* (Sheldrick, 2008 ▶); program(s) used to refine structure: *SHELXL97* (Sheldrick, 2008 ▶); molecular graphics: *ORTEP-3 for Windows* (Farrugia, 1997 ▶) and *PLATON* (Spek, 2009 ▶); software used to prepare material for publication: *WinGX* (Farrugia, 1999 ▶) and *PLATON*.

## Supplementary Material

Crystal structure: contains datablocks global, I. DOI: 10.1107/S1600536810036512/wm2399sup1.cif

Structure factors: contains datablocks I. DOI: 10.1107/S1600536810036512/wm2399Isup2.hkl

Additional supplementary materials:  crystallographic information; 3D view; checkCIF report

## Figures and Tables

**Table 1:** Hydrogen-bond geometry (Å, °) *Cg* is the centroid of the C14–C19 phenyl ring.

*D*—H⋯*A*	*D*—H	H⋯*A*	*D*⋯*A*	*D*—H⋯*A*
C10—H10*C*⋯O5^i^	0.96	2.678	3.452 (3)	138
C4—H4⋯*Cg*^i^	0.93	2.74	3.493 (3)	139

Symmetry code: (i) 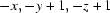.

**Fig. 1.**
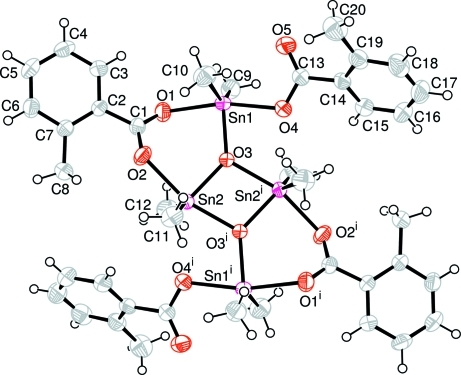
View of the molecular structure of the title compound with the atom numbering scheme. The displacement ellipsoids are drawn at the 50% probability level. H-atoms are shown as small spheres of arbitrary radius. [Symmetry code: (i) -x + 1, -y, -z + 1.]

## supplementary crystallographic information

### Comment

Recently we have reported the synthesis and crystal structure of a distannoxane (Danish *et al.*, 2009). In continuation of our interest in tin chemistry, the title compound (I), (Fig. 1) has been synthesized and structurally characterized. The crystal structure of bis(1,1,3,3-tetramethyl-1,3-dibenzoatodistannoxane) (Amini *et al.*, 2002) has also been published which is related to the title compound.

In the title molecule, both independent Sn atoms adopt distorted trigonal-bipyramidal coordination spheres with basal planes A (C9/C10/O3) and B (C11/C12/O3). The Sn—C and Sn—O bond lengths are lying in a range of 2.090 (2)–2.104 (3) Å and 2.0241 (14)–2.2561 (15) Å, respectively. The symmetry-related central four membered ring C (Sn2/O3/Sn2^i^/O3^i^; symmetry code: i = -*x* + 1, -*y*, -*z* + 1) is planar. The dihedral angles between A/C, B/C and A/B are 72.76 (4), 85.40 (6) and 57.54 (8)°, respectively. In (I), the 2-methylbenzoato ligands are not planar. In the *endo* ligand, group D (C2—C8) [r.m.s deviation of 0.0082 Å] is oriented at a dihedral angle of 32.9 (2)° with the carboxylato group E (O1/C1/O2). Whereas the *exo* ligand, group F (C14—C20) [r.m.s deviation of 0.0115 Å] is oriented at a dihedral angle of 45.3 (2)° with the carboxylato group G (O4/C13/O5). The dihedral angles between C/D and C/F are 5.43 (11) and 59.50 (7)°, respectively. There exist a weak C—H···O and a C—H···π interaction (Table 1). The packing mainly features van der Waals interactions between the molecules.

### Experimental

The sodium salt of *o*-toluic acid (0.316 g, 2.0 mmol) was suspended in 25 ml dry methanol in a 100 ml round-bottom flask. To this suspension, dimethyl tin dichloride (0.22 g, 1 mmol), dissolved in 25 ml dry methanol, was added dropwise with constant stirring at room temperature. The reaction mixture was refluxed for 6 h. Filtration was carried out to remove sodium chloride formed during reaction. Colorless prism of (I) were obtained after 48 h from the filtrate. M.p 486 K; yield: 71%.

### Refinement

Some low angle reflections were omitted from the refinement due to the beam stop effect. The H-atoms were positioned geometrically (C–H = 0.93–0.96 Å) and refined as riding with *U*_iso_(H) = *xU*_eq_(C), where *x* = 1.5 for methyl and *x* = 1.2 for aryl H-atoms.

### Crystal data

**Table:**

[Sn_4_(CH_3_)_8_(C_8_H_7_O_2_)_4_O_2_]	*Z* = 1
*M**_r_* = 1167.66	*F*(000) = 572
Triclinic, *P*1	*D*_x_ = 1.737 Mg m^−^^3^
Hall symbol: -P 1	Mo *K*α radiation, λ = 0.71073 Å
*a* = 10.0413 (2) Å	Cell parameters from 4786 reflections
*b* = 10.1280 (2) Å	θ = 2.1–28.3°
*c* = 12.0910 (3) Å	µ = 2.26 mm^−^^1^
α = 83.300 (1)°	*T* = 296 K
β = 72.850 (2)°	Prism, colorless
γ = 71.876 (1)°	0.30 × 0.26 × 0.23 mm
*V* = 1116.24 (4) Å^3^	

### Data collection

**Table:**

Bruker APEXII CCD diffractometer	5470 independent reflections
Radiation source: fine-focus sealed tube	4786 reflections with *I* > 2σ(*I*)
graphite	*R*_int_ = 0.024
Detector resolution: 7.50 pixels mm^-1^	θ_max_ = 28.3°, θ_min_ = 2.1°
ω scans	*h* = −13→13
Absorption correction: multi-scan (*SADABS*; Bruker, 2005)	*k* = −13→13
*T*_min_ = 0.514, *T*_max_ = 0.593	*l* = −16→16
18376 measured reflections	

### Refinement

**Table:**

Refinement on *F*^2^	Primary atom site location: structure-invariant direct methods
Least-squares matrix: full	Secondary atom site location: difference Fourier map
*R*[*F*^2^ > 2σ(*F*^2^)] = 0.018	Hydrogen site location: inferred from neighbouring sites
*wR*(*F*^2^) = 0.049	H-atom parameters constrained
*S* = 1.09	*w* = 1/[σ^2^(*F*_o_^2^) + (0.0194*P*)^2^ + 0.3314*P*] where *P* = (*F*_o_^2^ + 2*F*_c_^2^)/3
5470 reflections	(Δ/σ)_max_ = 0.002
250 parameters	Δρ_max_ = 0.43 e Å^−^^3^
0 restraints	Δρ_min_ = −0.40 e Å^−^^3^

### Special details

**Table:**

Geometry. Bond distances, angles *etc*. have been calculated using the rounded fractional coordinates. All su's are estimated from the variances of the (full) variance-covariance matrix. The cell e.s.d.'s are taken into account in the estimation of distances, angles and torsion angles
Refinement. Refinement of *F*^2^ against ALL reflections. The weighted *R*-factor *wR* and goodness of fit *S* are based on *F*^2^, conventional *R*-factors *R* are based on *F*, with *F* set to zero for negative *F*^2^. The threshold expression of *F*^2^ > σ(*F*^2^) is used only for calculating *R*-factors(gt) *etc*. and is not relevant to the choice of reflections for refinement. *R*-factors based on *F*^2^ are statistically about twice as large as those based on *F*, and *R*- factors based on ALL data will be even larger.

### Fractional atomic coordinates and isotropic or equivalent isotropic displacement parameters (Å^2^)

**Table:**

	*x*	*y*	*z*	*U*_iso_*/*U*_eq_	
Sn1	0.22041 (1)	0.28397 (1)	0.53408 (1)	0.0339 (1)	
Sn2	0.57013 (1)	0.05882 (1)	0.58448 (1)	0.0328 (1)	
O1	0.26212 (16)	0.33067 (15)	0.69668 (12)	0.0441 (5)	
O2	0.50311 (16)	0.27701 (15)	0.64565 (14)	0.0488 (5)	
O3	0.39865 (14)	0.11621 (13)	0.51399 (12)	0.0374 (4)	
O4	0.20722 (17)	0.18882 (15)	0.38766 (13)	0.0474 (5)	
O5	0.0386 (2)	0.38219 (17)	0.36273 (15)	0.0601 (6)	
C1	0.3791 (2)	0.3503 (2)	0.69953 (17)	0.0364 (6)	
C2	0.3696 (2)	0.46856 (19)	0.76660 (16)	0.0341 (6)	
C3	0.2507 (2)	0.5860 (2)	0.77069 (19)	0.0432 (7)	
C4	0.2381 (3)	0.7032 (2)	0.8260 (2)	0.0497 (7)	
C5	0.3430 (3)	0.7029 (2)	0.8776 (2)	0.0495 (7)	
C6	0.4602 (3)	0.5859 (2)	0.87427 (19)	0.0462 (7)	
C7	0.4771 (2)	0.4666 (2)	0.81952 (17)	0.0376 (6)	
C8	0.6062 (3)	0.3421 (2)	0.8215 (2)	0.0528 (8)	
C9	0.0303 (2)	0.2487 (3)	0.6466 (2)	0.0514 (8)	
C10	0.2897 (3)	0.4523 (2)	0.4481 (2)	0.0565 (8)	
C11	0.7553 (3)	0.1127 (2)	0.4805 (2)	0.0510 (8)	
C12	0.4894 (3)	−0.0106 (3)	0.7538 (2)	0.0572 (8)	
C13	0.1215 (2)	0.2684 (2)	0.32976 (18)	0.0423 (7)	
C14	0.1306 (2)	0.2099 (2)	0.21784 (18)	0.0415 (6)	
C15	0.1326 (3)	0.0718 (2)	0.2182 (2)	0.0503 (7)	
C16	0.1385 (3)	0.0126 (3)	0.1193 (2)	0.0597 (9)	
C17	0.1461 (3)	0.0905 (3)	0.0183 (2)	0.0656 (10)	
C18	0.1470 (3)	0.2263 (3)	0.0163 (2)	0.0603 (9)	
C19	0.1369 (2)	0.2902 (3)	0.11569 (19)	0.0477 (7)	
C20	0.1334 (3)	0.4405 (3)	0.1102 (3)	0.0668 (10)	
H3	0.17942	0.58560	0.73612	0.0518*	
H4	0.15890	0.78179	0.82819	0.0596*	
H5	0.33508	0.78146	0.91482	0.0594*	
H6	0.53017	0.58723	0.90992	0.0555*	
H8A	0.66157	0.36151	0.86691	0.0792*	
H8B	0.66647	0.32205	0.74390	0.0792*	
H8C	0.57295	0.26342	0.85508	0.0792*	
H9A	0.05583	0.17731	0.70272	0.0771*	
H9B	−0.02124	0.21985	0.60307	0.0771*	
H9C	−0.03061	0.33286	0.68556	0.0771*	
H10A	0.30365	0.44761	0.36649	0.0847*	
H10B	0.38001	0.44916	0.46205	0.0847*	
H10C	0.21741	0.53757	0.47635	0.0847*	
H11A	0.73019	0.17370	0.41799	0.0764*	
H11B	0.83035	0.03018	0.44956	0.0764*	
H11C	0.78978	0.15895	0.52634	0.0764*	
H12A	0.54898	−0.00324	0.80065	0.0858*	
H12B	0.49124	−0.10588	0.75313	0.0858*	
H12C	0.39110	0.04533	0.78523	0.0858*	
H15	0.12990	0.01860	0.28650	0.0604*	
H16	0.13734	−0.07909	0.12108	0.0716*	
H17	0.15067	0.05150	−0.04918	0.0787*	
H18	0.15457	0.27700	−0.05339	0.0724*	
H20A	0.19466	0.45179	0.15394	0.1002*	
H20B	0.03524	0.49670	0.14206	0.1002*	
H20C	0.16794	0.46896	0.03100	0.1002*	

### Atomic displacement parameters (Å^2^)

**Table:**

	*U*^11^	*U*^22^	*U*^33^	*U*^12^	*U*^13^	*U*^23^
Sn1	0.0307 (1)	0.0301 (1)	0.0392 (1)	−0.0034 (1)	−0.0120 (1)	−0.0034 (1)
Sn2	0.0304 (1)	0.0345 (1)	0.0344 (1)	−0.0063 (1)	−0.0115 (1)	−0.0060 (1)
O1	0.0397 (8)	0.0516 (9)	0.0440 (8)	−0.0135 (7)	−0.0113 (6)	−0.0126 (7)
O2	0.0406 (8)	0.0430 (8)	0.0617 (10)	−0.0078 (6)	−0.0088 (7)	−0.0237 (7)
O3	0.0355 (7)	0.0316 (6)	0.0467 (8)	−0.0004 (5)	−0.0203 (6)	−0.0099 (6)
O4	0.0507 (9)	0.0438 (8)	0.0483 (9)	0.0001 (7)	−0.0264 (7)	−0.0083 (6)
O5	0.0612 (11)	0.0534 (10)	0.0574 (10)	0.0092 (8)	−0.0259 (8)	−0.0158 (8)
C1	0.0383 (10)	0.0380 (10)	0.0323 (10)	−0.0095 (8)	−0.0095 (8)	−0.0038 (8)
C2	0.0351 (10)	0.0324 (9)	0.0333 (10)	−0.0083 (8)	−0.0074 (7)	−0.0047 (7)
C3	0.0392 (11)	0.0399 (11)	0.0470 (12)	−0.0030 (9)	−0.0133 (9)	−0.0076 (9)
C4	0.0497 (13)	0.0345 (10)	0.0544 (14)	0.0006 (9)	−0.0096 (10)	−0.0084 (9)
C5	0.0609 (14)	0.0365 (11)	0.0484 (13)	−0.0140 (10)	−0.0070 (10)	−0.0126 (9)
C6	0.0482 (12)	0.0490 (12)	0.0455 (12)	−0.0154 (10)	−0.0138 (9)	−0.0109 (9)
C7	0.0379 (10)	0.0366 (10)	0.0361 (10)	−0.0080 (8)	−0.0080 (8)	−0.0061 (8)
C8	0.0499 (13)	0.0507 (13)	0.0588 (14)	−0.0017 (10)	−0.0263 (11)	−0.0115 (11)
C9	0.0386 (12)	0.0555 (13)	0.0587 (14)	−0.0151 (10)	−0.0073 (10)	−0.0082 (11)
C10	0.0666 (16)	0.0438 (12)	0.0581 (15)	−0.0186 (11)	−0.0158 (12)	0.0060 (10)
C11	0.0465 (13)	0.0465 (12)	0.0556 (14)	−0.0168 (10)	−0.0036 (10)	−0.0028 (10)
C12	0.0550 (14)	0.0631 (15)	0.0442 (13)	−0.0122 (12)	−0.0058 (10)	0.0005 (11)
C13	0.0404 (11)	0.0436 (11)	0.0436 (12)	−0.0074 (9)	−0.0160 (9)	−0.0051 (9)
C14	0.0344 (10)	0.0462 (11)	0.0417 (11)	−0.0025 (9)	−0.0147 (8)	−0.0072 (9)
C15	0.0517 (13)	0.0475 (12)	0.0502 (13)	−0.0039 (10)	−0.0215 (10)	−0.0051 (10)
C16	0.0617 (16)	0.0532 (14)	0.0621 (16)	−0.0041 (12)	−0.0204 (12)	−0.0199 (12)
C17	0.0602 (16)	0.0784 (19)	0.0527 (15)	−0.0040 (14)	−0.0143 (12)	−0.0287 (14)
C18	0.0558 (15)	0.0817 (19)	0.0375 (12)	−0.0121 (13)	−0.0120 (10)	−0.0026 (12)
C19	0.0395 (11)	0.0577 (13)	0.0439 (12)	−0.0092 (10)	−0.0132 (9)	−0.0031 (10)
C20	0.0742 (18)	0.0673 (17)	0.0639 (17)	−0.0287 (14)	−0.0237 (14)	0.0143 (13)

### Geometric parameters (Å, °)

**Table:**

Sn1—O1	2.2561 (15)	C18—C19	1.394 (3)
Sn1—O3	2.0241 (14)	C19—C20	1.506 (4)
Sn1—O4	2.1671 (16)	C3—H3	0.9300
Sn1—C9	2.098 (2)	C4—H4	0.9300
Sn1—C10	2.090 (2)	C5—H5	0.9300
Sn2—O2	2.2463 (15)	C6—H6	0.9300
Sn2—O3	2.0387 (15)	C8—H8A	0.9600
Sn2—C11	2.104 (3)	C8—H8B	0.9600
Sn2—C12	2.094 (2)	C8—H8C	0.9600
Sn2—Sn2^i^	3.2993 (2)	C9—H9A	0.9600
Sn2—O3^i^	2.1412 (13)	C9—H9B	0.9600
O1—C1	1.261 (3)	C9—H9C	0.9600
O2—C1	1.259 (3)	C10—H10A	0.9600
O4—C13	1.293 (3)	C10—H10B	0.9600
O5—C13	1.223 (3)	C10—H10C	0.9600
C1—C2	1.487 (3)	C11—H11A	0.9600
C2—C3	1.392 (3)	C11—H11B	0.9600
C2—C7	1.402 (3)	C11—H11C	0.9600
C3—C4	1.384 (3)	C12—H12A	0.9600
C4—C5	1.373 (4)	C12—H12B	0.9600
C5—C6	1.381 (3)	C12—H12C	0.9600
C6—C7	1.386 (3)	C15—H15	0.9300
C7—C8	1.505 (3)	C16—H16	0.9300
C13—C14	1.507 (3)	C17—H17	0.9300
C14—C15	1.392 (3)	C18—H18	0.9300
C14—C19	1.394 (3)	C20—H20A	0.9600
C15—C16	1.377 (3)	C20—H20B	0.9600
C16—C17	1.370 (4)	C20—H20C	0.9600
C17—C18	1.376 (4)		
			
Sn1···O5^ii^	3.6541 (17)	C1···H8C	2.9700
Sn2···C13^i^	4.045 (2)	C1···H10B	2.9300
Sn1···H11B^i^	3.3500	C1···H8B	3.0100
Sn2···H15^i^	3.6200	C3···H20B^ii^	3.0900
O1···Sn2	3.4765 (15)	C4···H11A^iv^	3.0300
O1···O3	2.9929 (19)	C5···H8A^iii^	3.0700
O1···C9	2.937 (3)	C5···H12B^v^	2.8300
O1···C10	3.080 (3)	C6···H8A^iii^	3.0500
O2···C8	2.842 (3)	C6···H6^iii^	2.9800
O2···Sn1	3.4633 (17)	C7···H10A^iv^	2.9100
O2···O3	2.990 (2)	C8···H10A^iv^	3.0600
O2···C11	2.888 (3)	C9···H11B^i^	2.9400
O2···C12	3.077 (3)	C10···H8B^iv^	3.1000
O3···O3^i^	2.5685 (19)	C13···H10A	3.0900
O3···C10	3.314 (2)	C13···H20A	2.7400
O3···C12	3.294 (3)	C14···H4^ii^	3.0900
O3···O4	2.676 (2)	C14···H3^ii^	3.0900
O3···O1	2.9929 (19)	C15···H4^ii^	3.0300
O3···O2	2.990 (2)	C15···H12A^i^	3.0000
O3···C12^i^	3.287 (3)	C16···H4^ii^	3.0000
O3···C1	3.374 (2)	C17···H4^ii^	3.0400
O3···C11^i^	3.147 (3)	C19···H3^ii^	3.0900
O4···Sn2^i^	2.8567 (16)	H3···O1	2.5100
O4···O3	2.676 (2)	H3···O5^ii^	2.7100
O4···C10	3.245 (3)	H3···C14^ii^	3.0900
O4···C11^i^	3.243 (2)	H3···C19^ii^	3.0900
O4···C9	3.134 (3)	H3···H20B^ii^	2.5400
O4···C12^i^	3.113 (3)	H4···C14^ii^	3.0900
O5···C10	3.278 (4)	H4···C15^ii^	3.0300
O5···Sn1^ii^	3.6541 (17)	H4···C16^ii^	3.0000
O5···C20	2.965 (4)	H4···C17^ii^	3.0400
O1···H3	2.5100	H5···H12B^v^	2.5200
O2···H8B	2.4600	H6···H8A	2.2900
O2···H8C	2.8000	H6···C6^iii^	2.9800
O2···H11C	2.7600	H6···H20A^iv^	2.5500
O4···H12B^i^	2.9100	H8A···H6	2.2900
O4···H9B	2.8900	H8A···C5^iii^	3.0700
O4···H11B^i^	2.8300	H8A···C6^iii^	3.0500
O4···H15	2.6400	H8B···O2	2.4600
O5···H20A	2.7000	H8B···C1	3.0100
O5···H20B	2.7900	H8B···C10^iv^	3.1000
O5···H3^ii^	2.7100	H8C···O2	2.8000
O5···H9C^ii^	2.8600	H8C···C1	2.9700
O5···H10C^ii^	2.6800	H9B···H11C^vi^	2.6000
C3···C19^ii^	3.581 (3)	H9C···O5^ii^	2.8600
C6···C6^iii^	3.499 (3)	H10A···C13	3.0900
C7···C10^iv^	3.569 (3)	H10A···C7^iv^	2.9100
C8···O2	2.842 (3)	H10A···C8^iv^	3.0600
C9···O4	3.134 (3)	H10B···C1	2.9300
C9···O1	2.937 (3)	H10C···O5^ii^	2.6800
C10···C7^iv^	3.569 (3)	H11A···C4^iv^	3.0300
C10···C1	3.379 (3)	H11B···Sn1^i^	3.3500
C10···O4	3.245 (3)	H11B···O4^i^	2.8300
C10···C13	3.518 (3)	H11B···C9^i^	2.9400
C10···O5	3.278 (4)	H11C···H9B^vii^	2.6000
C10···O3	3.314 (2)	H12A···C15^i^	3.0000
C10···O1	3.080 (3)	H12B···C5^viii^	2.8300
C11···O2	2.888 (3)	H12B···H5^viii^	2.5200
C11···O4^i^	3.242 (2)	H12B···O4^i^	2.9100
C11···Sn1^i^	3.970 (2)	H15···O4	2.6400
C11···O3^i^	3.147 (3)	H15···Sn2^i^	3.6200
C12···O3^i^	3.287 (3)	H18···H20C	2.3500
C12···C1	3.517 (3)	H20A···O5	2.7000
C12···O4^i^	3.113 (3)	H20A···C13	2.7400
C12···O3	3.294 (3)	H20A···H6^iv^	2.5500
C12···O2	3.077 (3)	H20B···O5	2.7900
C13···Sn2^i^	4.044 (2)	H20B···C3^ii^	3.0900
C19···C3^ii^	3.581 (3)	H20B···H3^ii^	2.5400
C20···O5	2.965 (4)	H20C···H18	2.3500
			
O1—Sn1—O3	88.56 (6)	C14—C19—C20	122.4 (2)
O1—Sn1—O4	166.35 (5)	C18—C19—C20	120.1 (2)
O1—Sn1—C9	84.75 (8)	C2—C3—H3	120.00
O1—Sn1—C10	90.16 (8)	C4—C3—H3	120.00
O3—Sn1—O4	79.26 (6)	C3—C4—H4	120.00
O3—Sn1—C9	114.11 (9)	C5—C4—H4	120.00
O3—Sn1—C10	107.30 (8)	C4—C5—H5	120.00
O4—Sn1—C9	94.55 (8)	C6—C5—H5	120.00
O4—Sn1—C10	99.29 (8)	C5—C6—H6	119.00
C9—Sn1—C10	138.08 (11)	C7—C6—H6	119.00
O2—Sn2—O3	88.35 (6)	C7—C8—H8A	109.00
O2—Sn2—C11	83.12 (7)	C7—C8—H8B	109.00
O2—Sn2—C12	90.24 (9)	C7—C8—H8C	109.00
Sn2^i^—Sn2—O2	126.86 (4)	H8A—C8—H8B	109.00
O2—Sn2—O3^i^	162.12 (6)	H8A—C8—H8C	109.00
O3—Sn2—C11	113.89 (8)	H8B—C8—H8C	110.00
O3—Sn2—C12	105.70 (10)	Sn1—C9—H9A	109.00
Sn2^i^—Sn2—O3	38.99 (4)	Sn1—C9—H9B	109.00
O3—Sn2—O3^i^	75.78 (5)	Sn1—C9—H9C	109.00
C11—Sn2—C12	139.56 (11)	H9A—C9—H9B	109.00
Sn2^i^—Sn2—C11	108.32 (7)	H9A—C9—H9C	109.00
O3^i^—Sn2—C11	95.66 (7)	H9B—C9—H9C	109.00
Sn2^i^—Sn2—C12	107.47 (8)	Sn1—C10—H10A	110.00
O3^i^—Sn2—C12	101.83 (9)	Sn1—C10—H10B	109.00
Sn2^i^—Sn2—O3^i^	36.80 (4)	Sn1—C10—H10C	109.00
Sn1—O1—C1	123.85 (13)	H10A—C10—H10B	109.00
Sn2—O2—C1	129.00 (14)	H10A—C10—H10C	109.00
Sn1—O3—Sn2	132.78 (7)	H10B—C10—H10C	109.00
Sn1—O3—Sn2^i^	122.76 (7)	Sn2—C11—H11A	109.00
Sn2—O3—Sn2^i^	104.22 (6)	Sn2—C11—H11B	110.00
Sn1—O4—C13	115.13 (12)	Sn2—C11—H11C	110.00
O1—C1—O2	123.10 (19)	H11A—C11—H11B	109.00
O1—C1—C2	118.30 (18)	H11A—C11—H11C	109.00
O2—C1—C2	118.57 (19)	H11B—C11—H11C	110.00
C1—C2—C3	116.73 (19)	Sn2—C12—H12A	109.00
C1—C2—C7	122.57 (18)	Sn2—C12—H12B	109.00
C3—C2—C7	120.64 (18)	Sn2—C12—H12C	109.00
C2—C3—C4	120.3 (2)	H12A—C12—H12B	109.00
C3—C4—C5	119.7 (2)	H12A—C12—H12C	110.00
C4—C5—C6	120.0 (2)	H12B—C12—H12C	109.00
C5—C6—C7	122.2 (3)	C14—C15—H15	119.00
C2—C7—C6	117.3 (2)	C16—C15—H15	119.00
C2—C7—C8	123.55 (19)	C15—C16—H16	120.00
C6—C7—C8	119.2 (2)	C17—C16—H16	120.00
O4—C13—O5	123.1 (2)	C16—C17—H17	120.00
O4—C13—C14	114.83 (17)	C18—C17—H17	120.00
O5—C13—C14	122.09 (19)	C17—C18—H18	119.00
C13—C14—C15	118.28 (18)	C19—C18—H18	119.00
C13—C14—C19	121.75 (19)	C19—C20—H20A	110.00
C15—C14—C19	120.0 (2)	C19—C20—H20B	109.00
C14—C15—C16	121.2 (2)	C19—C20—H20C	109.00
C15—C16—C17	119.2 (3)	H20A—C20—H20B	109.00
C16—C17—C18	120.2 (2)	H20A—C20—H20C	109.00
C17—C18—C19	121.9 (2)	H20B—C20—H20C	109.00
C14—C19—C18	117.5 (2)		
			
O3—Sn1—O1—C1	57.73 (15)	C12—Sn2—Sn2^i^—C12^i^	−180.00 (12)
C9—Sn1—O1—C1	172.10 (17)	O3^i^—Sn2—Sn2^i^—O3	−179.98 (13)
C10—Sn1—O1—C1	−49.57 (17)	O3—Sn2—O3^i^—Sn1^i^	174.92 (9)
O1—Sn1—O3—Sn2	−16.38 (10)	O3—Sn2—O3^i^—Sn2^i^	−0.03 (14)
O1—Sn1—O3—Sn2^i^	156.91 (8)	C11—Sn2—O3^i^—Sn1^i^	61.67 (10)
O4—Sn1—O3—Sn2	169.83 (11)	C11—Sn2—O3^i^—Sn2^i^	−113.25 (8)
O4—Sn1—O3—Sn2^i^	−16.88 (8)	C12—Sn2—O3^i^—Sn1^i^	−81.66 (12)
C9—Sn1—O3—Sn2	−99.97 (11)	C12—Sn2—O3^i^—Sn2^i^	103.42 (10)
C9—Sn1—O3—Sn2^i^	73.32 (10)	Sn1—O1—C1—O2	−42.7 (3)
C10—Sn1—O3—Sn2	73.34 (12)	Sn1—O1—C1—C2	135.22 (15)
C10—Sn1—O3—Sn2^i^	−113.38 (10)	Sn2—O2—C1—O1	−23.7 (3)
O3—Sn1—O4—C13	−163.73 (16)	Sn2—O2—C1—C2	158.41 (13)
C9—Sn1—O4—C13	82.56 (17)	Sn1—O4—C13—O5	−9.5 (3)
C10—Sn1—O4—C13	−57.73 (17)	Sn1—O4—C13—C14	170.80 (14)
O3—Sn2—O2—C1	49.17 (18)	O1—C1—C2—C3	−32.3 (3)
C11—Sn2—O2—C1	163.47 (19)	O1—C1—C2—C7	150.43 (19)
C12—Sn2—O2—C1	−56.53 (19)	O2—C1—C2—C3	145.7 (2)
Sn2^i^—Sn2—O2—C1	55.71 (19)	O2—C1—C2—C7	−31.6 (3)
O2—Sn2—O3—Sn1	−14.15 (10)	C1—C2—C3—C4	−176.5 (2)
O2—Sn2—O3—Sn2^i^	171.68 (7)	C7—C2—C3—C4	0.8 (3)
C11—Sn2—O3—Sn1	−95.88 (11)	C1—C2—C7—C6	176.54 (19)
C11—Sn2—O3—Sn2^i^	89.95 (8)	C1—C2—C7—C8	−4.7 (3)
C12—Sn2—O3—Sn1	75.64 (12)	C3—C2—C7—C6	−0.6 (3)
C12—Sn2—O3—Sn2^i^	−98.54 (9)	C3—C2—C7—C8	178.2 (2)
Sn2^i^—Sn2—O3—Sn1	174.18 (14)	C2—C3—C4—C5	−0.4 (3)
O3^i^—Sn2—O3—Sn1	174.18 (11)	C3—C4—C5—C6	−0.1 (4)
O3^i^—Sn2—O3—Sn2^i^	0.03 (13)	C4—C5—C6—C7	0.3 (4)
O2—Sn2—Sn2^i^—O3	−10.42 (8)	C5—C6—C7—C2	0.1 (3)
O2—Sn2—Sn2^i^—O2^i^	180.00 (8)	C5—C6—C7—C8	−178.8 (2)
O2—Sn2—Sn2^i^—O3^i^	169.58 (8)	O4—C13—C14—C15	45.0 (3)
O2—Sn2—Sn2^i^—C11^i^	−84.82 (8)	O4—C13—C14—C19	−135.0 (2)
O2—Sn2—Sn2^i^—C12^i^	76.02 (10)	O5—C13—C14—C15	−134.7 (3)
O3—Sn2—Sn2^i^—O2^i^	−169.58 (8)	O5—C13—C14—C19	45.3 (3)
O3—Sn2—Sn2^i^—O3^i^	179.98 (13)	C13—C14—C15—C16	179.0 (3)
O3—Sn2—Sn2^i^—C11^i^	−74.40 (9)	C19—C14—C15—C16	−1.0 (4)
O3—Sn2—Sn2^i^—C12^i^	86.45 (11)	C13—C14—C19—C18	179.2 (2)
C11—Sn2—Sn2^i^—O3	−105.61 (9)	C13—C14—C19—C20	−0.7 (4)
C11—Sn2—Sn2^i^—O2^i^	84.81 (8)	C15—C14—C19—C18	−0.8 (4)
C11—Sn2—Sn2^i^—O3^i^	74.39 (9)	C15—C14—C19—C20	179.3 (3)
C11—Sn2—Sn2^i^—C11^i^	180.00 (9)	C14—C15—C16—C17	1.6 (5)
C11—Sn2—Sn2^i^—C12^i^	−19.16 (11)	C15—C16—C17—C18	−0.4 (5)
C12—Sn2—Sn2^i^—O3	93.56 (11)	C16—C17—C18—C19	−1.5 (5)
C12—Sn2—Sn2^i^—O2^i^	−76.02 (10)	C17—C18—C19—C14	2.1 (4)
C12—Sn2—Sn2^i^—O3^i^	−86.45 (11)	C17—C18—C19—C20	−178.0 (3)
C12—Sn2—Sn2^i^—C11^i^	19.16 (11)		

Symmetry codes: (i) −*x*+1, −*y*, −*z*+1; (ii) −*x*, −*y*+1, −*z*+1; (iii) −*x*+1, −*y*+1, −*z*+2; (iv) −*x*+1, −*y*+1, −*z*+1; (v) *x*, *y*+1, *z*; (vi) *x*−1, *y*, *z*; (vii) *x*+1, *y*, *z*; (viii) *x*, *y*−1, *z*.

### Hydrogen-bond geometry (Å, °)

**Table:**

Cg is the centroid of the C14–C19 phenyl ring.

**Table:**

*D*—H···*A*	*D*—H	H···*A*	*D*···*A*	*D*—H···*A*
C10—H10C···O5^ii^	0.96	2.678	3.452 (3)	138
C4—H4···Cg^ii^	0.93	2.74	3.493 (3)	139

Symmetry codes: (ii) −*x*, −*y*+1, −*z*+1.
